# Around the Hospitals

**Published:** 1897-08-28

**Authors:** 


### AROUND THE HOSPITALS.

[Notice to the Managers of Institutions.—Special space is now reserved for the insertion of notices of hospital meetings and festivals. The Editor requests that all notices may reach The Hospital Office, 28 & 29, Southampton Street, Strand, London, W.C., at least one week before the date of each meeting.]

**Queen's Hospital, Birmingham.**—The governors of this hospital, although their income amounted to some £460 more in 1896 than it did in 1895, managed to reduce their expenditure last year by about £140. This is the more noticeable because both the in and the out-patients show an increase in numbers, viz., in-patients, 120 (1895, 1,989; 1896, 2,109); and out-patients, 312 (1895, 28,508; 1896, 28,820). We therefore arrive at the fact that, after deducting 2s. 6d. for each out-patient (the amount the hospital authorities estimate an out-patient costs them), each in-patient in 1896 cost £2 9s. 1d., a saving per patient of 4s. 9d. when compared with 1895. As each in-patient was resident in the hospital 21·5 days in 1896 against 22·3 days in 1895, the saving in the cost of a patient a day amounts to about three-halfpence (*i.e.*, 2s. 3½d. against 2s. 4⅝d.) There have been several changes in the staff during the year under review. First of all, in order to render the work in the out-patients' department less difficult, two additional medical officers, viz., one physician and one surgeon, have been appointed for the treatment of casualties and out-patients. Secondly, the senior surgeon, Mr. J. St. S. Wilders, who had been connected with the hospital for forty years, has resigned his post owing to ill-health, and has been elected one of the consulting surgeons. Next, a new matron, Miss Elkington, has been appointed, in place of the late Miss Mary Cadbury, whose sad death took place in September last. And, finally, Mr. F. G. Messiter, one of the house physicians, died from typhoid fever in November. Two structural improvements have been made during the year, one the provision of an exterior iron staircase for use in case of fire, and the other improvements in the ventilation of the kitchen, the cost of both of which was kindly borne by two gentlemen interested in the hospital. We are glad to see that this hospital also has adopted the uniform system of accounts, but we must repeat what we said about the General Hospital, that we fail to see why the subscriptions received from workpeople should not be shown separately, seeing what importance Birmingham rightly attaches to the support given to its hospitals by the working men.

**Bolton Infirmary.**—Owing to an outbreak of scarlet fever in 1896, the children's wards at the Bolton Infirmary were closed for over six weeks, and, as a consequence, there were 180 fewer in-patients treated, 1,141 having been treated in the year ended February, 1896, and 961 in 1896-7. The out-patients (3,794) and casualties (1,865) show increases of 182 and 876 respectively, however, whilst the home-patients (548) show a decrease of 51 when compared with 1895-6. On an average, 37 males, 21 females, and 25 children—or 83 patients in all—were resident in the hospital each day in 1896-7. With the aid of the Cotton Famine Districts Fund 74 patients were sent to convalescent hospitals during the year, most of them to Southport. The receipts for the year amounted to £5,450, and the expenditure to £5,670, the difference being supplied from the "donations" account. Amongst the receipts is £2,200, received from the Hospital Saturday Fund, the highest sum yet contributed by the workpeople of Bolton. Before we leave the accounts we must express our surprise that the uniform system has not been adopted by this institution. Here, as at Leeds and Hereford, practically all the details necessary are given in the report, the only apparent trouble being the rearranging and regrouping of the items in the form suggested in the uniform system of accounts. The chief building operation undertaken during the year was the erection of a new operating theatre to replace the old one, which had become unsuitable for the requirements of modern surgery and was very inefficiently lighted.

